# The Relationships among Trait Anxiety, State Anxiety and the Goal Performance of Penalty Shoot-Out by University Soccer Players

**DOI:** 10.1371/journal.pone.0035727

**Published:** 2012-04-23

**Authors:** Masami Horikawa, Akihiro Yagi

**Affiliations:** Department of Psychological Sciences, Kwansei Gakuin University, Nishinomiya, Japan; Federal University of Rio de Janeiro, Brazil

## Abstract

The present study examined how the level of trait anxiety, which is a personality characteristic, influences state anxiety and penalty shoot-out performance under pressure by instruction. The high and low trait anxiety groups were selected by using Spielberger's Trait Anxiety Scale, with trait anxiety scores, and control and pressure conditions manipulated by instructions. The participants were two groups of eight university male soccer players. They individually performed 20 shots from the penalty shoot-out point, aiming at the top right and top left corner areas in the soccer goal. Each condition had 10 trials in a within-subject design. The dependent measures comprised the number of successful goals and the state anxiety scores under each instructional condition. The result showed a significant main effect of instruction. State anxiety scores increased more and the number of successful goals decreased more in high trait anxiety groups than in low trait anxiety groups under pressure instructional condition. These findings suggest that players with higher trait anxiety scores tend to experience increased state anxiety under a pressure-laden condition, and higher state anxiety interferes with goal performance.

## Introduction

The penalty shoot-out is used to break tied games in soccer tournaments. It is also a one-to-one challenge between a goalkeeper and the penalty-taker. In general, penalty-takers are considered to be at an advantage, therefore, they feel anxiety and tension under pressure to convert the penalty and consequently often fail to perform as expected. Even world-famous and exceptionally skilled soccer players have failed in penalty shoot-outs during international matches such as the World Cup, hence, the penalty shoot-out is a special situation for soccer players [1]–[4]. Reportedly, psychological loads such as stress and tension affect the outcome of penalty kicks [1]. Using video analyses, Jordet and Hartman [3] found that avoidance behavior, such as preparing the shot quickly, occurred more frequently with the shot where a miss leads to loss. In a study that examined the effect of goalkeeper, goalkeeper distracted players' gaze behavior, and reduced their shooting accuracy [5]–[6].

The pressure for success is one of the key factors affecting players' performance in sport and usually increases their anxiety. According to Spielberger [7], there are two kinds of anxieties; state anxiety and trait anxiety. State anxiety reflects a transitory emotional state or a condition that is characterized by subjective, consciously perceived feelings of tension and apprehension, and heightened autonomic nervous system activity. It may fluctuate and can vary in intensity. In contrast, trait anxiety refers to a general tendency to respond with anxiety to perceived threats in the environment, and is a relatively stable characteristic of an individual. An individual with higher trait anxiety feels more threats in many situations than someone with low trait anxiety. In addition, anticipated failure or threats to self-esteem can be more devastating than threats to physiological condition. To evaluate two different types of anxieties, Spielberger developed the State-Trait Anxiety Inventory (THE STAI). Each form has 20 items using four-point Likert-scales, with total scale scores ranging from 20 to 80 [8].

An individual with higher trait anxiety score tends to have higher state anxiety score. Scores of state anxiety are rated high in circumstances where an individual feels the situation to be threatening irrespective of the objective danger. State anxiety scores should be low in non-stressful situations or in situations where an existing danger is not perceived as threatening [9]. So as both high and low levels of state anxiety can interfere with performance, it is considered that the relationship between state anxiety and performance would show an inverted-U relationship [10]. Although each researcher [10]–[11] accounted for the arousal and performance, using different dimension of the model, all agree that performance improves as arousal level increases. However, if it becomes too high, performance deteriorates.

A number of studies have provided evidence of the relationship between competitive trait and state anxiety in competitive situations, Using the Sport Competition Anxiety Test (THE SCAT) [12] and the Competitive State Anxiety Inventory (THE CSAI, CSAI-2) [13], both of which have shown to be more sensitive scales in the sports context than THE STAI. The literature still lacks consistent results on the relationship between trait anxiety and performance. Jones and his colleagues stated that direction that represented the labeling of internal state was a better indicator of performance than intensity alone [14]–[16]. Their findings showed that high trait anxiety performers with positive expectations, and low trait anxiety performers with negative expectations reported their anxiety as more facilitative than low trait anxiety performers with positive expectations and high trait anxiety performers with negative expectation [14]. Furthermore, Elite performers interpreted state anxiety as being more facilitative to performance than the non-elite performers, and anxiety intensity levels of performers with debilitative interpretations were higher than those with facilitative in non-elite performer, whereas no such differences were in the elite performers [16].

One of the theoretical models accounting for both the debilitating and facilitating effects of anxiety is processing efficiency theory [17]–[18]. According to Eysenck and Calvo [17]–[18], the level of performance is “determined” by state anxiety, which is a product of trait anxiety and situational stress. Worry is a component of state anxiety responsible for effects of anxiety on performance effectiveness and efficiency. Effectiveness refers to the quality of task performance, whereas efficiency refers to the amount of cognitive effort invested to attain a given performance effectiveness. Worry consumes some of the processing resources in working memory, and worry also increases motivation to minimize the aversive anxiety state. Potential performance would be impaired, if cognitive effort and additional resources are not compensated for in order to cope with worry. Furthermore, a positive cognitive interpretation of anxiety would improve performance but a negative one would impair performance. High trait anxiety individuals interpret anxiety less positively compared with low trait anxiety individuals, therefore, they tend to experience more deficits in both performance efficiency and effectiveness.

Recently, Wilson and his colleagues [5]–[6], [18]–[20] have examined a performance efficiency theory using various measures in sport setting such as simulated archery and penalty shoot-out. When these and other researchers [5]–[6], [19], [21]–[23] manipulated anxiety level with a pressure-laden instruction, the players' performance deteriorated as shown in the reduction of shooting accuracy and an increase of response time under high threat condition.

The purpose of the present study was to examine how the level of trait anxiety affects state anxiety and the penalty shoot-out performance under pressure by experimental instruction. For this purpose, experimental groups were selected by trait anxiety scores, and the experimental conditions were manipulated by specific instruction. The number of successful goals and scores of state anxiety were measured. Our hypotheses were that: (1) the pressure elicited by the experimental instruction would adversely affect performance, (2) the pressure would exacerbate the level of state anxiety, and (3) both the performance and state anxiety of the high trait anxiety group would be affected most negatively by the pressure instruction.

## Methods

### Ethics

The study was approved by the faculty meeting of the Department of Psychological Sciences of Kwansei Gakuin University. We followed the ethical standards of the American Psychological Association, and through this, informed consents were obtained.

### Screening Test

Fifty nine male soccer players (age: 18–22 years, *M* = 20.3 years; years of playing soccer: 6–17 years, *M* = 11.9 years) were recruited from a university soccer club to participate in the screening test, all of whom signed an informed consent form. The Japanese version of the STAI (trait) [24] was used to assign participants to one of two groups (either high or low trait anxiety group), while they also reported their ages, years of playing soccer, kicking leg, and an evaluation of the skill level in their team. The mean score of their trait anxiety was 43.1(*SD* = 8.67), and the criterion for inclusion in the experimental group was above or below 1 *SD* from this mean score. This selection method resulted in 16 players. The 16 players were divided into two groups of high and low trait anxieties on the basis of their STAI scores, and two groups were formed respectively as uniformly as possible in terms of their years' playing soccer, kicking legs, and skill levels. The mean score of the high trait anxiety group (*n* = 8) was 54 (range: 50–60) while that of the low trait anxiety group (*n* = 8) was 31.63 (range: 25–37). The *t*-test showed a significant difference between the two groups (*t*(14) = 12.92, *p*<.05).

### Experimental Participants

The participants were 16 undergraduate male soccer players (age: 18–22 years, *M* = 20.5 years; years of playing soccer: 10–18 years, *M* = 12.6 years), who were in good health. 14 participants were right-footed and 2 were left-footed. All participants signed informed consent including a detailed explanation of the purpose and procedures of the experiment.

### Experimental Design and Setting

The experimental design of the present study was a 2 (trait anxiety group: high, low)×2 (instructional condition: control, pressure) design with repeated measures across the instructional conditions. The dependent variables were state anxiety, as measured by the STAI and the number of successful goal performance (ranging from 0 to 10 for each instructional condition).

The experiment was conducted on a soccer field (105 m long×68 m wide). The penalty shoot-out point was 11 meters from the center of the goal line. The soccer goal (2.44 m high×7.32 m wide) was divided into 12 blocks using plastic tapes with three horizontal lines and four vertical lines. Each area was 0.81 meters high and 1.83 meters wide.

### Procedure

Each participant was told to shoot 10 penalties from the penalty shoot-out point to the goal under each of the two instructions. In the present study, we deliberately avoided the use of a goalkeeper, in order to eliminate the influence of goalkeeper performance on results [5]–[6]. Participants were told to shoot at the top right and top left corners respectively in the soccer goal. Prior to the experiment, we asked several university soccer players excluding the participants to shoot at all target areas and asked them which areas they felt difficult to kick accurately. Based on their responses, we determined these target areas.

The shooting conditions were manipulated by the experimenter instructions: a control condition (control) and a pressure condition (pressure). Each condition involving 10 trial shots and the experiment was conducted over two days. The control and the pressure condition were assigned on the first and second days, respectively. All participants performed 20 trials in total, at trial intervals of 20 seconds and with the two target areas for shooting counterbalanced. Under the control condition, all participants were instructed that they were free to shoot at the start whistle. Under the pressure condition, they were firmly told to increase their successful goal score from the control condition level. In the pressure condition, we used several manipulations to induce anxiety, namely by a higher level of verbal instructional pressure to shoot successfully and to be more competitive. While some studies reinforced performer's shooting outcome [5]–[6], [19], [21]–[23], we manipulated the antecedent condition of shooting by giving them “pressure instructions” and citing how others performed previously, as we have found the effectiveness of using performance standards in a Japanese team sport. Participants were told of their performance of the control condition, and then they were firmly told to increase their successful goal score from that of the first day's shooting (under the control condition). At the same time, they were shown slightly inflated information showing how other successful goal scores on the second day (under pressure instruction) had shot more goals than on their first day (under control instruction).The first author provided all instructions and experimental debriefings.

### Measures

Before the beginning of the experiment on the first day, all participants responded to the state anxiety items of the STAI to establish a baseline. On both days, they initially warmed up using a soccer ball for five minutes, after the specific instruction. Just prior to shooting, they retook the STAI. The difference scores of the STAI-state anxiety of all participants were then calculated by subtracting the baseline state anxiety scores from those of the post experimental instruction, with the participants' performances measured in terms of the number of successful goals in 10 trials.

## Results

Table 1 shows the means and *SD*s of trait anxiety and state anxiety as well as number of successful goals of low and high trait anxiety groups.

**Table 1:** Means and *SDs* of Trait Anxiety, State Anxiety and Number of successful goals.

Measures	Condition		Group	
	low-anxiety		high-anxiety	
	Control	Pressure	Control	Pressure
Trait Anxiety Score	31.63 (4.17)		54.00 (3.12)	
State Anxiety Score	37.25 (4.84)	37.75 (5.20)	41.25 (5.92)	44.88 (5.47)
Number of successful goals	6.25 (1.71)	5.50 (1.66)	4.13 (1.17)	3.75 (0.66)

The value in a parenthesis shows *SD*.

**Figure 1:**
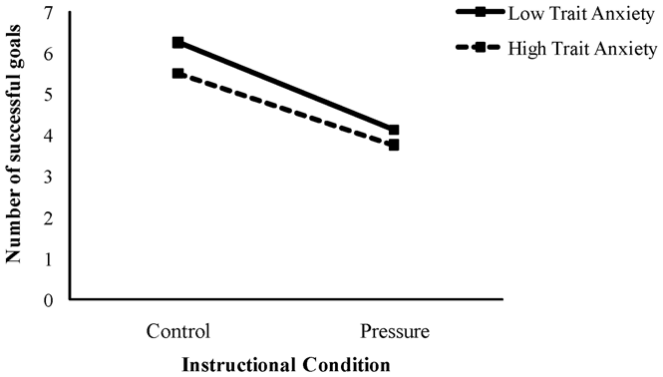
Mean successful goals for each trait anxiety group and instructional condition. The solid and dotted lines are respectively the Low and High Trait Anxiety Groups.

Figure 1 shows the mean numbers of successful goals for high and low trait anxiety groups across the two instructional conditions. To examine the effects of the trait anxiety group and the instructional condition, we performed a 2 (high and low trait anxiety groups) by 2 (control and pressure instructions) repeated measures ANOVA on the number of successful goals. The main effect of the instructional condition was significant, *F* (1, 14) = 27.68, *p*<.01, *η^2^* = 0.66. A further Bonferroni test revealed that the number of successful goals under the pressure condition was significantly lower than the control condition (*p*<.01). The successful goals results across the two conditions indicated deterioration of performance under the pressure condition by the experimental instruction. These results support our hypothesis (1).

**Figure 2:**
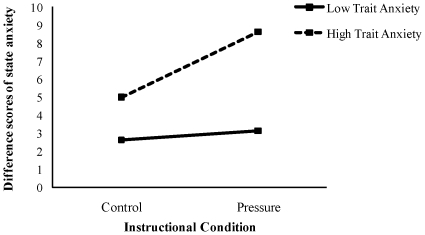
Mean difference scores of state anxiety for each trait anxiety group and instructional condition. The solid and dotted lines are respectively the Low and High Trait Anxiety Groups.

Figure 2 shows the mean difference scores of state anxiety for each trait anxiety group and instructional condition. To examine the effect of the trait anxiety group and instructional condition, we conducted a 2 (high and low trait anxiety groups) by 2 (control and pressure instructions) ANOVA with repeated measures across the instructional conditions. The main effect of the trait anxiety group was significant, *F* (1, 14) = 5.68, *p*<.05, *η^2^* = 0.29, as was the main effect of the instructional condition, *F* (1, 14) = 5.75, *p*<.05, *η^2^* = 0.29. The interaction effect of the trait anxiety group and the instructional condition was not significant and the effect size was small, *F* (1, 14) = 3.29, *p*<.09, *η^2^* = 0.19.

A further Bonferroni test revealed that the mean state anxiety score of the high trait anxiety group was significantly higher than that of the low trait anxiety group (*p*<.05), and that the mean state anxiety score under the pressure condition was significantly higher than that of the control condition (*p*<.01). A simple effects analysis for the trait anxiety group showed a significant difference for the pressure condition, *F*(1, 28) = 8.72, *p*<.01, *η^2^* = 0.24. And a simple effects analysis for the high trait anxiety group was not significant for the pressure condition, *F* (1, 28) = 3.79, *p*<.06, *η^2^* = 0.12. The state anxiety score results across the two conditions indicated increases of anxiety level under the pressure condition by the experimental instruction. These results support our hypothesis (2). Overall, the results of successful goal and state anxiety score across the two groups and condition indicated deterioration of goal performance and an increase of anxiety level for high trait anxiety group under the pressure condition. These results support our hypothesis (3).

## Discussion

A number of research studies have investigated the relationships between trait anxiety, state anxiety, and performance with a consistent result that anxiety induced by the pressure for success influences performance positively or negatively. In the competitive sports situation that required a success result, it is important to reduce, as much as possible, debilitating effects of anxiety from performance. A number of factors contribute to increased anxiety. In the present study, we manipulated anxiety level with a pressure-laden instruction, and examined whether the level of trait anxiety influences state anxiety and penalty shoot-out performance in the experimental setting.

The previous studies [5]–[6], [19], [21]–[23] showed deterioration of performance by the pressure-laden instruction. Our results also showed that competitive situation by the instruction, that is, the pressure for success, increased the anxiety level and produced a deterioration of goal performance. These results support our hypotheses (1) and (2), and they are also consistent with those of previous studies [5]–[6], [19], [21]–[23]. Performance of both high and low trait anxiety groups deteriorated under the pressure-laden instruction, although all participants in the present study had played soccer for over 10 years and were top level players among Japanese university soccer players. Although Jones [16] reported that elite performer interpreted anxiety as more facilitative to performance, the present study found that pressure causes a deterioration of performance. It would be important for coaches and trainers to assess how soccer players cope with the pressure of performing the penalty shoot-out.

As we confirmed that the pressure for success influenced state anxiety and goal performance, we now discuss whether the level of trait anxiety affects the level of state anxiety and goal performance. Based on Spielberger's statement [7], [9] and processing efficiency theory [17]–[18], we predicted that the level of state anxiety score would be highest for the high trait anxiety group under pressure instruction. In light of Spielberger's model [7], [9] and the inverted-U model [10]–[11], we predicted that goal performance of this group would deteriorate most negatively. Hypothesis (3) was partially supported.

We now look into the results of the high trait anxiety group in detail. According to processing efficiency theory [17]–[18], the scores of state anxiety indicates consumption of performance efficiency and the number of successful goals indicates performance effectiveness. State anxiety was likely determined by the interaction of trait anxiety and situational stress induced by negative interpretations of the pressure instruction. Under these circumstances, both performance efficiency and performance effectiveness were impaired. High trait anxiety group may have interpreted their anxiety as being negative and debilitative to performance, consequently their performance deteriorated. The present results lend an empirical support to Jones and Swain's findings [15].

Turning attention to the low trait anxiety group, we found that their goal performance under pressure condition deteriorated more than those under control condition, but their state anxiety score hardly increased under pressure condition. We question why their performance was impaired without relation to state anxiety level under pressure condition. From Spielberger's statement [7], [9] and processing efficiency theory [17]–[18], it is considered that low trait anxiety group interpreted their anxiety more positively than high trait anxiety group. In view of Jones and his colleague's findings [14]–[16], it is likely that their motivation did not increase by state anxiety. Based on inverted-U models [10]–[11] and processing efficiency theory [17]–[18], it is possible that the players' anxiety level was too low and not at an optimal level to perform a penalty-shoot out under pressure condition, or their cognitive effort and additional resources by their motivation did not compensate for poor performance, thus ultimately their performance was impaired.

There are three limitations of the present study. First, the measurement timing for state anxiety and performance was non-synchronous. Although the level of state anxiety was lower at the beginning of the experiment, it was likely to change as the shooting trials progressed. Each shooting outcome might affect participants' anxiety and motivation. Previous studies reported avoidance behavior after negative shots [3], and faster first fixations of anxious penalty-takers [5]–[6]. Thus, we may be able to assess the relationship between state anxiety and performance more accurately by measuring state anxiety before each trial, performing a single trial, or examining participants' behavior during trial intervals.

Secondly, this study used the number of successful goals as an index of effectiveness. Shots were kicked into two target areas. These shots became centralized we recommend measuring distance and direction from the target area of unsuccessful goals and were within the goalkeeper's reach [5]–[6]. In this way, we may evaluate performance effectiveness more comprehensively.

Finally, a third limitation concerns the absence of a goalkeeper in the penalty shoot out of the present experiment. The interaction of goalkeeper influences penalty-taker's anxiety actually, as the previous researches showed distraction of attention and negative influences on performance [5]–[6]. In this regard, the ecological validity of the current experimental shooting situation was compromised without using a goalkeeper.

To conclude, the results shows, for the high trait anxiety group, the state anxiety score increased more and the successful goals decreased more in the high trait anxiety groups than in the low trait anxiety groups under pressure condition. Furthermore, for the low trait anxiety group, the successful goals deteriorated, although the state anxiety score increased little under the pressure condition. Our findings suggest that higher trait anxiety tends to have higher state anxiety and higher state anxiety interferes with goal performance. These results have implications for the development of a coaching program for university soccer players. The results offer empirical support to Spielberger's [7], [9], Eysenck et al.'s [17]–[18] and Jones et al.'s [14]–[16] models accounting for the relationship between state and trait anxieties and sport performance.
